# Impact of Female Sex on Outcomes of Patients Undergoing Thoracic Endovascular Aortic Aneurysm Repair: A Ten-Year Retrospective Nationwide Study in France

**DOI:** 10.3390/jcm11082253

**Published:** 2022-04-18

**Authors:** Fabien Lareyre, Juliette Raffort, Christian-Alexander Behrendt, Arindam Chaudhuri, Cong Duy Lê, Roxane Fabre, Christian Pradier, Laurent Bailly

**Affiliations:** 1 Department of Vascular Surgery, Hospital of Antibes Juan-les-Pins, 06600 Antibes, France; duy02a205@gmail.com; 2 Université Côte d’Azur, Inserm U1065, C3M, 06204 Nice, France; juliette.raffort@hotmail.fr; 3 3IA Institute, Université Côte d’Azur, 06204 Nice, France; 4 Department of Clinical Biochemistry, University Hospital of Nice, 06001 Nice, France; 5 Research Group GermanVasc, Department of Vascular Medicine, University Heart and Vascular Centre UKE Hamburg, University Medical Centre Hamburg-Eppendorf, 20246 Hamburg, Germany; ch.behrendt@uke.de; 6 Bedfordshire-Milton Keynes Vascular Centre, Bedfordshire Hospitals NHS Foundation Trust, Bedford MK42 9DJ, UK; a.chaudhuri@ntlworld.com; 7 CoBTeK Lab, Université Côte d’Azur, 06001 Nice, France; fabre.r@chu-nice.fr; 8 Public Health Department, University Hospital of Nice, Université Côte d’Azur, 06001 Nice, France; pradier.c@chu-nice.fr (C.P.); bailly.l@chu-nice.fr (L.B.); 9 UR2CA—Unité de Recherche Clinique Côte d’Azur, Université Côte d’Azur, 06000 Nice, France

**Keywords:** thoracic aortic aneurysm, thoracic endovascular repair, TEVAR, outcomes, nationwide study, sex

## Abstract

The impact of sex on the outcomes of patients with cardiovascular disease is still incompletely understood. The aim of this nationwide multicenter observational study was to investigate the impact of sex on post-operative outcomes in patients undergoing thoracic endovascular aortic repair (TEVAR) for intact thoracic aortic aneurysm (iTAA). The French National Health Insurance Information System was searched to identify these patients over a ten-year retrospective period. Post-operative outcomes, 30-day and overall mortality were recorded. Among the 7383 patients included (5521 men and 1862 women), females were significantly older than males (66.8 vs. 64.8 years, *p* < 0.001). They were less frequently diagnosed with cardiovascular comorbidities. Post-operatively, women had less frequently respiratory (10.9 vs. 13.7%, *p* = 0.002) as well as cardiac complications (34.3 vs. 37.3%, *p* = 0.023), but they had more frequently arterial complications (52.8 vs. 49.8%, *p* = 0.024). There was no significant difference on overall mortality for a mean follow-up of 2.2 years (26.9 vs. 27.6%, *p* = 0.58). In the multivariable regression model, female sex was not associated with 30-day or overall mortality. Although women had a favorable comorbidity profile, the short-term and long-term survival was similar. The significantly higher rate of arterial complications suggests that women may be at higher risk of access-vessel-related complications.

## 1. Introduction

There is growing evidence for differences between sexes in terms of cardiovascular disease prevalence, clinical symptoms, and outcomes [1,2,3]. Several meta-analyses and large registry studies have suggested that women may be treated differently and are at higher risk of peri-operative mortality and morbidity following vascular interventions in the aorta and peripheral arteries [4,5,6]. Thoracic endovascular aortic repair (TEVAR) has become a well-established alternative to treat aortic diseases including descending thoracic aortic aneurysm (TAA), complicated type B aortic dissection or traumatic aortic injury [7,8,9,10,11,12]. This minimally invasive technique can be used to treat intact and ruptured TAA (iTAA/rTAAA), offering repair to patients unfit or at high risk for traditional open surgery [13,14,15,16,17], whilst also minimizing risks of stroke and paraplegia [8,18]. While the short and longer-term outcomes of patients following TEVAR have been reported [8,9,13,14,15,17,19,20,21,22,23,24,25,26,27], the impact of sex is still poorly known. 

TAA is a life-threatening disease associated with high morbidity and mortality rates [8,28]. The prevalence of TAA is lower in women [29]. Epidemiologic and clinical research often involve a higher proportion of men [8]. As a consequence, current guidelines contain very few sex-specific recommendations [8,30]. It is suggested that TAA growth rates are higher in women [31] and that acute aortic syndromes may occur at smaller aneurysm sizes in women than men [28,32]. It is estimated that the size of the thoracic aorta is approximately 10% smaller in women [30]. However, data currently available do not allow to conclude whether lower thresholds for TAA repair should be systematically considered in women [30]. A better understanding of risk profiles between sexes may help to improve care provided to patients, particularly with the increased diagnoses of such pathologies with modern imaging techniques [29] and the presence of TAA as a comorbid condition along with abdominal aortic aneurysm (AAA) (particularly as noted in women [33]). The aim of this ten-year nationwide multicenter study was to investigate the impact of sex on post-operative outcomes in patients undergoing TEVAR for iTAA. 

## 2. Material and Methods

### 2.1. Study Design and Data Collection

This nationwide multicenter retrospective study was based on the French National Health Insurance Information System. Data have been extracted from the “Programme de Médicalisation du Système d’Information Français (PMSI)”, which compiles standardized data on all patients admitted into all French public and private hospitals and provides exhaustive information on all surgical interventions in France. The study complied with the principles laid down in the Declaration of Helsinki. The protocol was approved by the Institutional Review Board of the University Hospital of Nice and by the French National Health Information System (Système National des Données de Santé, SNDS). The study was conducted in accordance with the French National Health Data Institute regulation (Institut National des Données de Santé, INDS). Informed consent was waived in accordance with the French National Health Data Institute regulation and guidelines on the use of routinely collected research data. Data were extracted from anonymous discharge reports completed and coded at the end of each hospital stay. The database has been previously used and described [34,35]. 

The database was searched to identify all patients who were admitted for the first time (index treatment) in any public or private hospital for TEVAR for intact TAA in France between 1 January 2011 and 31 December 2020. TEVAR was defined according to the Common Classification of Medical Acts (CCAM) using the appropriate procedure codes DGAF007 and DGLF003. The information was retrieved from standardized reports, which are obligatory and constitute the basis of hospital funding. Each report contains diagnoses and therapeutic procedures. A unique alphanumerical anonymous identifier identifies each patient, which allows follow-up of all inpatients after the first admission.

Data collected included age (in years), dichotomized sex, and comorbidities. Comorbidities were defined according to the International Classification of Diseases (ICD-10). The codes used were the following: arterial hypertension (I10), dyslipidæmia (E78.0 to E78.5), diabetes (E10, E11, E12, E13, E14), obesity (E65 and E66), smoking (F17), and congestive heart failure (I50, I11, I13, I09, I25, I42 and I43). Other general comorbidities were those of the Charlson comorbidity index score [36]. ICD codes matching each item for this index were extracted according to the algorithm developed by Quan et al. [37]. 

Events occurring within the hospital stay were collected. It included the occurrence of respiratory complications (pneumonia, need of mechanic respiratory assistance), vascular complications (major bleeding, red blood cell transfusion, arterial complications, arterial thrombosis, complications related to the thoracic aortic endograft), and cardiac complications or neurologic complications (stroke). The codes used to define post-operative complications are detailed in the Supplementary Material. Arterial complications were defined according to ICD-10 and included aortic aneurysm and dissection (I71), other aneurysm including aneurysm of lower extremity (I72), arterial embolism and thrombosis (I74), acquired arterio-venous fistula (I77.0), stenosis of artery (I77.1), and rupture of arteries (I77.2). We further collected data regarding in-hospital length of stay, in-hospital mortality, transfer to intensive care unit (ICU), and duration of ICU stay (as well as post-operative complications). For the follow-up, patients who had at least one re-admission at hospital after TEVAR recorded in the National French electronic health data were identified and included. 30-day, 90-day, and long-term overall mortality were defined as deaths recorded during a hospital stay that occurred after discharge from the first repair, whatever the cause. 

### 2.2. Statistical Analysis

Results for continuous data were expressed as means +/− standard deviation (SD) and as numbers with percentages for categorical data. Differences between men and women were analyzed using either Student’s *t*-test for quantitative variables or Chi-squared test for qualitative variables. A survival analysis was performed to identify factors associated with the risk of overall and 30-day mortality. The follow-up started from the day of the first hospital stay for TEVAR. Kaplan-Meier survival curve analysis and log-rank tests were used to analyze the risk of post-operative mortality by sex. To compare mortality, a multivariate-adjusted Cox proportional hazards regression model was performed to estimate adjusted hazard ratios (HR) and 95% confidence intervals (CI) on the outcomes, controlling for age, gender, history of cancer, acute coronary event, pneumonia or respiratory failure, and arterial complication during the in-hospital stay for TEVAR. 

The hypothesis of the proportional risk was evaluated according to the survival curves using the double negative logarithm function. The factors which did not comply with the hypothesis of the proportional risk were excluded from the model. A two-tailed *p*-value < 0.05 was considered as statistically significant. No correction for multiple hypothesis testing was applied. Statistical analysis was performed using SAS Enterprise 5.1 (SAS Institute, Cary, NC, USA).

## 3. Results

Over a ten-year retrospective period, 7383 patients underwent TEVAR for iTAA repair including 5521 men (74.8%) and 1862 women (25.2%) (Table 1). Females undergoing TEVAR were significantly older than males (66.8 vs. 64.8 years, *p* < 0.001). They were less frequently diagnosed with cardiovascular comorbidities including dyslipidemia (14.9 vs. 17.3%, *p* = 0.02), diabetes (8.6 vs. 10.6%, *p* = 0.012) and smoking (9.5 vs. 11.7%, *p* = 0.01). They had less frequently a past history of myocardial infarction (2.1 vs. 3.8%, *p* < 0.001). The proportion of chronic kidney disease (7.8 vs. 11.4%, *p* < 0.001) and chronic hepatic disease (1.6 vs. 2.4%, *p* = 0.03) amongst women was also lower. The mean Charlson comorbidity index was significantly lower in women (3.4 vs. 3.7, *p* < 0.001). 

The mean length of in-hospital stay did not significantly differ between women and men (16.7 vs. 16.2 days, *p* = 0.39) (Table 2). There was no difference on in-hospital mortality (11.6 vs. 11.4%, *p* = 0.82) or ICU stay (40.0 vs. 30.4%, *p* = 0.63). Regarding immediate post-operative complications, women had less frequently pneumonia (10.9 vs. 13.7%, *p* < 0.01) as well as cardiac complications (34.3 vs. 37.3%, *p* = 0.02). However, they had more frequently arterial complications (52.8 vs. 49.8%, *p* = 0.24) and more specifically arterial thrombosis (10.5 vs. 8.4%, *p* < 0.01). 

The 30-day and the 90-day mortality did not differ between women and men (10.1 vs. 9.5%, *p* = 0.46 and 13.5 vs. 13.4%, *p* = 0.89, respectively) (Table 2). For a mean follow-up of 2.2 years, the over-all mortality was similar between women and men (26.9 vs. 27.6%, *p* = 0.58). In multivariable regression model, age over 67 years was identified as a predictive factor of 30-day mortality (hazard ratio, HR = 1.51, 95% CI: 1.29–1.76, *p* < 0.001) (Table 3). Furthermore, the occurrence of cardiac and respiratory complications was associated with the risk of 30-day mortality. Regarding over-all mortality, age over 67 years was a risk factor (HR = 1.99, 95% CI: 1.82–2.19, *p* < 0.001) (Table 4). History of cancer, post-operative cardiac, and respiratory complications, as well as renal disease, were associated with the risk of overall mortality. In multi-variate regression models, female sex was not significantly associated with the risk of 30-day or overall mortality. 

## 4. Discussion

This nationwide multicenter cohort study using insurance claims of 7383 patients who underwent TEVAR for intact TAA over a ten-year period in France revealed that women were selected for treatment at higher age but with less comorbidities when compared with men. Although severe respiratory and cardiac complications were less frequent in women, the short-term and long-term survival was similar between sexes. Interestingly, the higher rate of arterial complications in women urges for further research.

We did not identify a significant difference regarding immediate in-hospital post-operative mortality between men and women. This is in accordance with a report from an international consortium of vascular registries (VASCUNET) involving 13 countries [9]. Among 3815 patients who underwent TEVAR for intact TAA, the peri-operative mortality rate was 4.5% in women and 4.4% in men, with no significant difference among sexes (*p* = 0.98) [9]. 

In our study, no significant difference between women and men was observed on 30-day, 90-day, or over-all mortality. A meta-analysis was recently performed to investigate sex specific differences on 30-day mortality [4]. It included 7 studies with 2758 women and 4674 men who underwent TEVAR [19,38,39,40,41,42,43] and found that 30-day mortality was significantly higher in women, with a pooled rate of 5%, compared to 3% in men (OR = 1.75, 95% CI: 1.29–2.38) [4]. Another review reported 30-day mortality rates between 0% to 6% in female and from 1.1% to 5.3% for male patients [39,40,44,45,46].

The impact of sex on long-term mortality is still poorly known. We did not observe a significant difference between men and women for a mean follow-up of 2.2 years. In a national report including 2574 patients (40% women) who underwent TEVAR for iTAA, the one-year mortality was found to be higher in women (9.8% vs. 6.3%, *p* < 0.01) [41]. However, other studies did not observe a significant difference for one-year mortality. In a cohort of 195 patients, the mortality rate in women was 15.2% vs. 16.8% (*p* = 0.84) [38]. In another study including 421 patients, the one-year mortality rate was 13.3% in women and 9.5% in male patients (*p* = 0.23) [39]. Note that it is possible that the small sizes of the cohorts may have limited the statistical power of the analysis. 

While we did not identify a significant impact of sex on in-hospital length of stay, a meta-analysis including 7432 patients found that the length of stay was longer in women, with a standardized mean difference of 0.3 days (95% confidence interval: 0.14–0.47) [4]. In our cohort, women were less likely to have post-operative complications including pneumonia or cardiac complications. This might be, at least partly, explained by the fact that they had significantly a lower proportion of cardiovascular risk factors including dyslipidemia, diabetes, smoking and past history of myocardial infarction. This is in accordance with other studies which found that at baseline, women had less frequently associated comorbidities such as coronary artery disease, chronic obstructive pulmonary disease or smoking habit [39,41]. Nevertheless, as suggested by Ulug et al., it is highly possible that coronary artery disease may be underdiagnosed in women as symptoms are often less typical, with higher frequency of non-obstructive coronary disease [47,48,49,50]. Several studies have suggested that women were potentially less likely to receive optimal treatment for coronary artery disease compared to men [3,50]. It is notable that in our cohort, women were older than men (67 vs. 64 years, *p* < 0.001). Another national report including 2574 patients who underwent TEVAR also reported that women were slightly older than men (73 vs. 72 years, *p* = 0.03) [41] but other studies did not identify a difference on age between women and men at the time of TEVAR [38,41]. The higher life expectancy in women should be taken in consideration to interpret the results [51]. Several studies observed higher risk of post-operative events in women including respiratory complications, major adverse events or higher length of stay in ICU [38,41]. Increased number of bleeding complications or increased need for blood transfusion in women have also been reported in several studies [38,39,40,41]. We found that women had significantly higher rates of arterial complications. Compared to men, women present anatomical differences, such as a smaller access vessel diameter [4,39]. As a consequence, it is not surprising that higher vascular access-related complications have been reported in women [38,39], with more frequent need of iliac artery exposure (18% vs. 7%; *p* < 0.001) [40] or conduit use for device delivery (24.4% vs. 6.0%, *p* < 0.001) [38,39], as also a higher incidence of procedure-related iliac artery rupture, particularly at TEVAR [52]. A multicenter observational cohort study including 887 patients confirmed these findings, with an overall access related complication rate within 30 days after TEVAR of 2.8% in women and 1.8% in men (*p* = 0.013) [53]. After adjustment for age, urgency, device diameter, introducer sheath, access vessel diameters, and access method, female gender was significantly associated with the risk of access complications (OR = 2.85; *p* = 0.038). Finally, no significant difference was observed on the occurrence of stroke between women and men, which is in accordance with current literature [4,38,39,40,41,42,54]. Multivariable analysis confirmed that female sex was not significantly associated with the risk of post-operative mortality in the French National ten-year retrospective cohort. We found that age over 67 years and the occurrence of post-operative cardiac complications were significantly associated with the risk of 30-day as well as long-term mortality. The German nationwide study investigating the outcomes of patients who underwent TEVAR or open repair for TAA identified rupture, increasing age, and higher comorbidity score as significantly associated with higher mortality (RR = 6.66, 5.33–8.25; 1.28, 1.17–1.40; and 1.06, 1.05–1.08, respectively) [43]. The retrospective cohort study using the Society for Vascular Surgery Vascular Quality Initiative (SVS-VQI) including 2574 patients who underwent TEVAR identified age, aortic size index, symptoms, and female sex as independently predictive of 30-day and long-term mortality [41]. Another database from 649 patients who underwent TEVAR confirmed increasing age as independent predictor of 30-day mortality, along with emergency procedure and iliac artery exposure. In this study, female sex was no longer a significant predictor after multivariable analysis [40]. 

This study presents some limitations. First, it is based on electronic administrative database and the results may depend on the coding system. The French National Health Information System uses standardized definitions, and data are audited annually by experts to control and minimize potential bias in the coding system. Second, the database allowed to follow or record patient data only during a hospital-stay. Nevertheless, it can be assumed that most of the patients presenting with severe or life-threatening disease may have been hospitalized. Finally, the database did not record detailed information on the symptoms, clinical presentation of patients as well as procedural characteristics such zones covered by TEVAR, type of endografts used, landing zones, use of debranching, approaches used for femoral artery access, or operative time. The database did not record information on the aneurysm characteristics such the size/diameter of the TAA, presence of blister or penetrating atherosclerotic ulcer. While we could record arterial complications and arterial thrombosis that occurred post-operatively, we could not have detailed information on all access-vessel related complications or re-intervention rate. 

## 5. Conclusions

This nationwide analysis of patients who underwent TEVAR for intact TAA in France over a recent ten-year period revealed interesting differences between sexes. Although women had a favorable comorbidity profile and exhibited respiratory and cardiac complications less often, the short-term and long-term survival was similar. In light of the higher life expectancy of women, this result may be interpreted with caution. Furthermore, the significantly higher rate of arterial complications confirmed previous evidence of higher access-vessel related complications and emphasizes a possible health problem and anatomical differences. 

## Figures and Tables

**Table 1:** Baseline Characteristics of patients who underwent endovascular thoracic aortic aneurysm repair (TEVAR). Results are expressed as mean +/− SD or *n* (%).

	Total *n* = 7383	Men *n* = 5521	Women *n* = 1862	*p* Value
Age (years)	64.8 ± 15.7	64.1 ± 15.7	66.8 ± 15.5	<0.001
Arterial hypertension	4322 (58.5)	3239 (58.7)	1083 (58.2)	0.70
Dyslipidæmia	1232 (16.7)	954 (17.3)	278 (14.9)	0.02
Diabetes	747 (10.1)	587 (10.6)	160 (8.6)	0.01
Smoking	819 (11.1)	643 (11.7)	176 (9.5)	<0.01
Obesity	873 (11.8)	676 (12.2)	197 (10.6)	0.06
Heart failure	946 (12.8)	729 (13.2)	217 (11.7)	0.08
Myocardial infarction	248 (3.4)	210 (3.8)	38 (2.1)	0.0003
Chronic respiratory disease	873 (11.8)	665 (12.0)	208 (11.2)	0.312
Renal disease	777 (10.5)	631 (11.4)	146 (7.8)	<0.001
Chronic hepatic disease	163 (2.2)	134 (2.4)	29 (1.6)	0.03
Stroke	278 (3.8)	201 (3.6)	77 (4.1)	0.33
Cancer	431 (5.8)	339 (6.1)	92 (4.9)	0.06
Charlson comorbidity index	3.7 ± 3.1	3.8 ± 3.2	3.4 ± 2.7	<0.001

**Table 2:** Outcomes of patients who underwent endovascular thoracic aortic aneurysm repair (TEVAR).

	Total *n* = 7383	Men *n* = 5521	Women *n* = 1862	*p* Value
In-hospital stay (days)	16.3 ± 19.3	16.2 ± 19.4	16.7 ± 19.0	0.39
In-hospital mortality	842 (11.4)	627 (11.4)	215 (11.6)	0.82
Transfer to intensive care unit	2255 (30.5)	1678 (30.4)	577 (40.0)	0.63
Need of mechanic respiratory assistance	3065 (41.5)	2309 (41.8)	756 (40.6)	0.36
Pneumonia	957 (13.0)	755 (13.7)	202 (10.9)	<0.01
Major bleeding	971 (13.2)	711 (12.9)	260 (14.0)	0.23
Red blood cell transfusion	421 (5.7)	308 (5.6)	113 (6.1)	0.43
Pericarditis	229 (3.1)	166 (3.0)	63 (3.4)	0.42
Cardiac complication	2696 (36.5)	2057 (37.3)	639 (34.3)	0.02
Arterial complication	3731 (50.5)	2748 (49.8)	983 (52.8)	0.02
Arterial thrombosis	659 (8.9)	464 (8.4)	195 (10.5)	<0.01
Surgical site infection	833 (11.3)	609 (11.0)	224 (12.0)	0.24
Complication on the aortic graft	599 (8.1)	454 (8.2)	145 (7.8)	0.55
Stroke	278 (3.8)	201 (3.6)	77 (4.1)	0.33
30-day mortality	713 (9.7)	525 (9.5)	188 (10.1)	0.46
90-day mortality	988 (13.4)	737 (13.4)	251 (13.5)	0.89
Mean follow-up (years)	2.2 ± 2.6	2.3 ± 2.6	2.0 ± 2.4	<0.001
Overall mortality	2023 (27.4)	1522 (27.6)	501 (26.9)	0.58

**Table 3:** Factors associated with 30-day mortality.

	Adjusted HR	CI 95%	*p* Value
Age over 67 (ref. <67)	1.51	1.29–1.76	<0.001
Cardiac complication	1.98	1.70–2.31	<0.001
Respiratory complication	1.37	1.17–1.60	<0.001
Female sex (ref. male sex)	1.04	0.88–1.23	0.65

HR: Hazards ratio. ref.: Reference.

**Table 4:** Factors associated with overall mortality.

	Adjusted HR	CI 95%	*p* Value
Age over 67 (ref. <67)	1.99	1.82–2.19	<0.001
Cancer	2.01	1.74–2.31	<0.001
Cardiac complication	1.66	1.51–1.81	<0.001
Renal disease	1.47	1.30–1.66	<0.001
Respiratory complication	1.28	1.16–1.40	<0.001
Female sex (ref. male sex)	1.05	0.95–1.16	0.34

HR: Hazards ratio. ref.: Reference.

## Data Availability

Not applicable.

## Supplementary Materials

The following supporting information can be downloaded at: https://www.mdpi.com/article/10.3390/jcm11082253/s1.
