# Supplementary material for: Impact of Female Sex on Outcomes of Patients Undergoing Thoracic Endovascular Aortic Aneurysm Repair: A Ten-Year Retrospective Nationwide Study in France
Source: J Clin Med. 2022 Apr 18;11(8):2253. doi: 10.3390/jcm11082253 (PMC9029404; doi:10.3390/jcm11082253)
Supplement: Supplementary file 1 [file jcm-11-02253-s001.zip › jcm-1632452-supplementary.pdf]

Supplemental data

The ICD-10 codes used to define post-operative complications were:

- Pneumonia: J12 -J14,J16-J22

- Major bleeding : T810, T811, T812

- Red blood cell transfusion : Z513

- Surgical site infection: T814, T818, T819

- Complication on the thoracic aortic graft : T823-T829 T856-T859

- Arterial complication: aortic aneurysm and dissection (I71), other aneurysm including aneurysm of lower extremity (I72), arterial embolism and thrombosis (I74), acquired arterio-venous fistula (I77.0), stenosis of artery (I77.1), rupture of artery (I77.2).

- Cardiac complications : acute coronary event and acute coronary syndrome (I20 to I24), cardiopulmorary affection (I26, J80-81) , arrhythmia (I44 to I49), cardiac failure (I50-I51), pericarditis (I30,I313 I322), endocarditis (I33, I38), myocarditis (I40)

The CCAM codes used were:

- Transfer to intensive care unit : YYYY0200, YYYY0150, YYYY0100

- Need of mechanic respiratory assistance: GLLD0020- GLLD021
